# Effects of aging on the skin and gill microbiota of farmed seabass and seabream

**DOI:** 10.1186/s42523-020-00072-2

**Published:** 2021-01-12

**Authors:** Daniela Rosado, Marcos Pérez-Losada, Ana Pereira, Ricardo Severino, Raquel Xavier

**Affiliations:** 1 CIBIO-InBIO, Centro de Investigação em Biodiversidade e Recursos Genéticos, Universidade do Porto, Campus Agrário de Vairão, Vairão, 4485-661 Porto, Portugal; 2 Computational Biology Institute, Department of Biostatistics and Bioinformatics, Milken Institute School of Public Health, George Washington University, Washington, DC, 20052-0066 USA; 3 Piscicultura Vale da Lama, Sapal do Vale da Lama, Odiáxere, 8600-258 Lagos, Portugal

**Keywords:** *Dicentrarchus labrax*, *Sparus aurata*, Bacteria, Sexual maturation, Microbiota

## Abstract

**Background:**

Important changes in microbial composition related to sexual maturation have been already reported in the gut of several vertebrates including mammals, amphibians and fish. Such changes in fish are linked to reproduction and growth during developmental stages, diet transitions and critical life events. We used amplicon (16S rRNA) high-throughput sequencing to characterize the skin and gill bacterial microbiota of farmed seabass and seabream belonging to three different developmental age groups: early and late juveniles and mature adults. We also assessed the impact of the surrounding estuarine water microbiota in shaping the fish skin and gill microbiota.

**Results:**

Microbial diversity, composition and predicted metabolic functions varied across fish maturity stages. Alpha-diversity in the seabass microbiota varied significantly between age groups and was higher in older fish. Conversely, in the seabream, no significant differences were found in alpha-diversity between age groups. Microbial structure varied significantly across age groups; moreover, high structural variation was also observed within groups. Different bacterial metabolic pathways were predicted to be enriched in the microbiota of both species. Finally, we found that the water microbiota was significantly distinct from the fish microbiota across all the studied age groups, although a high percentage of ASVs was shared with the skin and gill microbiotas.

**Conclusions:**

We report important microbial differences in composition and potential functionality across different ages of farmed seabass and seabream. These differences may be related to somatic growth and the onset of sexual maturation. Importantly, some of the inferred metabolic pathways could enhance the fish coping mechanisms during stressful conditions. Our results provide new evidence suggesting that growth and sexual maturation have an important role in shaping the microbiota of the fish external mucosae and highlight the importance of considering different life stages in microbiota studies.

**Supplementary Information:**

The online version contains supplementary material available at 10.1186/s42523-020-00072-2.

## Background

Research on animal microbial communities (microbiota) is growing exponentially as the link between microbiota and host health is strongly validated by emerging evidence [1–8]. Age-related fluctuations in the microbiota are well studied in humans and are considered as “natural, inevitable and benign” [9]. Critical microbial changes occur during infancy and old age, coinciding with stages when the immune system is also more fragile [9]. Results linking changes in the gut microbiota to reproduction and growth (e.g., monkeys, [10]) or disease resistance in early life stages (e.g., amphibians, [11]) have been also found in other vertebrates.

In piscine hosts, most of the microbiota studies related to the effects of age are focused on the gut [12–22]. Some of these studies showed that microbial communities in the surrounding waters influence the gut microbiota during early life stages, which becomes increasingly unique with age [17, 21]. Indeed, initial microbiota colonization in animals is highly dependent on the environment (e.g., [23–27]). Ecological factors, such as diet transitions (e.g., [21]) or critical life events (e.g., habitat transition, [20]), which in turn are intrinsically linked to sexual maturation, also play a major role in shaping the fish gut microbiota. Importantly, most studies testing the role of age on fish microbiota were cross-sectional and based on a single time point or a short time window (e.g., [16, 18, 19, 21, 22]). Thus, given the high susceptibility of the fish microbiota to environmental changes and the high interindividual microbiota variability (e.g., [28–30]), the compound effect of all these factors could be hard to interpret [31].

Fish skin and gills and their associated mucous and microbes form a natural physical and chemical barrier to pathogens [4, 32, 33]. Despite this protective role, little is known about potential host developmental effects on skin and gill microbiota. Filling out this knowledge gap is particularly important in fish farming, where diseases are a main concern causing high mortality rates (e.g., [34]). Two previous studies in wild reef fish comparing the gill [14] and skin [35] microbiota of juvenile and mature adult fish from several species, showed a general pattern of differentiation between life-stages with differences attributed to intraspecific niche partitioning [14, 35]. Additionally, increases in body weight were seen to be associated with an increase in the microbial structure (i.e., beta-diversity) of the skin and gill microbiota of wild rabbitfish [36].

The European seabass (*Dicentrarchus labrax*) and the gilthead seabream (*Sparus aurata*) are two of the most important farmed fish in Europe, with a global production of 191,003 tns and 185,980 tns, respectively, in 2016 [37]. The gilthead seabream is a protandric hermaphrodite, maturing first as males between years 1 and 2, with sex reversal occurring in the following 2–3 years [38–40]. The European seabass reaches sexual maturity between years 2 and 3 in males, and after year 3 in females [41–43]. Typically, in semi-extensive production systems, both fish are reared until they reach their first commercial size (18–24 months). However, demand for larger fish sizes has been increasing, meaning that both species can reach sexual maturity before harvest.

Here we used amplicon (16S rRNA) high-throughput sequencing to characterize the skin and gill bacterial microbiota of farmed seabass and seabream from different ages (juvenile stages and mature adults). Our main goal was to describe differences in composition, structure and potential metabolic functions in their microbiota. Additionally, we investigated the impact of the microbial communities present in the water column on the skin and gill microbiotas.

## Results

Skin, gill and water microbial samples from different fish age groups were collected simultaneously (same day and at approximately the same time) from separate ponds (Additional file 1). Three age cohorts were sampled for the seabass, which included fish in their 1st, 2nd and 3rd year of age; while two age cohorts were sampled for the seabream, which included fish in their 2nd and 3rd year of age. Since our sampling was non-invasive, to classify fishes into age groups we coupled available information from the literature [38–43] with weight and age of maturation estimates provided by the fish farm. The three seabass age cohorts were thus classified as early juveniles, late juveniles and mature adults, respectively; while the two seabream age cohorts were classified as juveniles and mature adults, respectively – see Materials and Methods section for more details. Differences in the average weight estimated for each age group at the beginning and end of our sampling showed a 245% growth for the seabass early juvenile group, an 83% growth for the late juvenile group and a 43% growth for the mature adult group. Similarly, a 143% growth was estimated for the seabream juveniles and a 16% growth for the mature adults. Descriptive analyses were performed for each age group separately and comparative statistical analyses were performed between age groups.

### Microbial diversity across age groups

#### Alpha-diversity

**Fig. 1:**
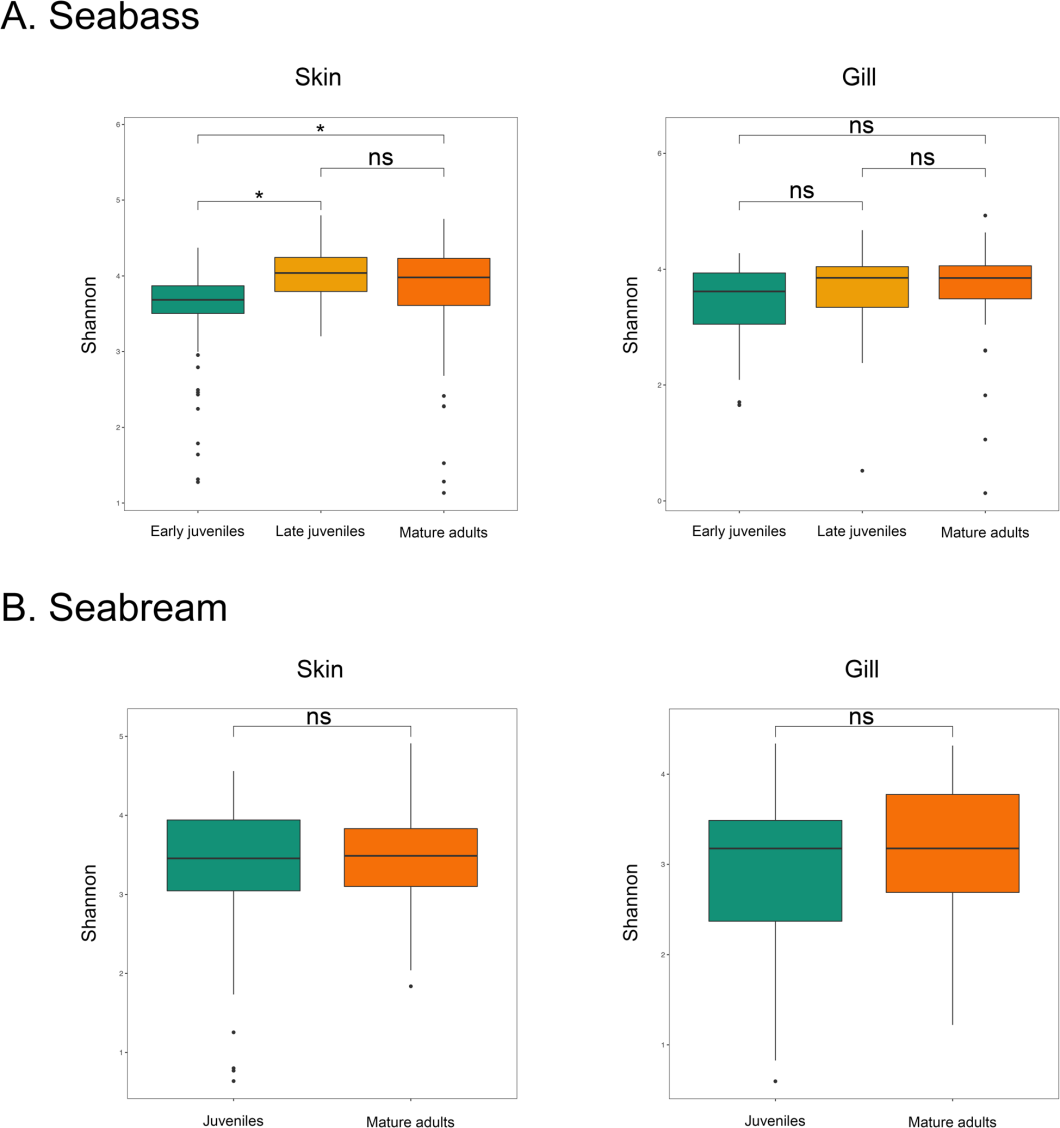
Mean values and standard deviations of Shannon alpha-diversity estimates plotted for the early juveniles/juveniles (green), late juveniles (yellow) and mature adults (orange) of the seabass *Dicentrarchus labrax* (**a**) and the seabream *Sparus aurata* (**b**) (*n* = 60 per species x age group x tissue). Pairwise comparisons of alpha-diversity were assessed using Linear Mixed Effect models with age groups as a fixed factor and sampling time as a random factor. Statistically significant differences are denoted with an asterisk and non statistically significant differences with “ns”

Microbial alpha-diversity was calculated using Shannon, Faith’s phylogenetic diversity (PD), ACE and Simpson 1-D indices. In general, the skin microbiota showed higher mean values for the alpha-diversity indices than the gill microbiota across all age groups in both fish species, except for the Simpson 1-D index in the late juveniles and adults of the seabass (Additional file 2). In seabass, the skin and gill microbiota of late juveniles and mature adult fish presented higher mean values of alpha-diversity than the microbiota of the early juveniles (Fig. 1a, Additional file 3). In seabream, skin and gill microbiotas showed similar alpha-diversity in both cohorts (Fig. 1b, Additional file 3). Linear Mixed Effects (LME) model analysis (diversity~age group + (1|sampling date)) showed most alpha-diversity estimates varied significantly between seabass age groups in both tissues. Pairwise comparisons between age groups in seabass showed significant (*p* < 0.05) differences in alpha-diversity for almost all the tests comparing early vs late juveniles and early juveniles vs mature adults (Table 1). Seabass late juvenile vs mature adult alpha-diversity comparisons were never significant (*p* > 0.05, Table 1) for both fish mucosae. In the seabream, only the Shannon and PD indices of the gill microbiota varied significantly between juveniles and mature adults (*p* < 0.04, Table 1).

**Table 1:** Mean alpha-diversity values, and alpha- and beta-diversity comparisons for the skin and gill microbiota of the different age groups of seabass *Dicentrarchus labrax* and seabream *Sparus aurata* (*n* = 60 per species x age group x tissue)

	Seabass								Seabream			
	Skin				Gill				Skin		Gill	
Alpha-diversity mean values	EJ		LJ	MA	EJ		LJ	MA	J	MA	J	MA
Shannon	3.5 ± 1		4 ± 0.4	3.8 ± 1	3.5 ± 1		3.6 ± 1	3.7 ± 1	3.3 ± 1	3.4 ± 1	2.9 ± 1	3.2 ± 1
PD	20 ± 7		28 ± 11	27 ± 9	19 ± 6		22 ± 8	23 ± 9	19 ± 9	18 ± 8	13 ± 6	15 ± 6
ACE	164 ± 66		239 ± 106	226 ± 83	138 ± 52		159 ± 69	175 ± 73	155 ± 77	145 ± 71	99 ± 49	110 ± 54
Simpson 1-D	18 ± 8		25 ± 8	23 ± 11	20 ± 11		23 ± 10	24 ± 11	15 ± 10	16 ± 9	11 ± 8	14 ± 10
Alpha-diversity comparisons	Overall	EJ vs LJ	LJ vs MA	EJ vs MA	Overall	EJ vs LJ	LJ vs MA	EJ vs MA	J vs MA		J vs MA	
Shannon	**16 (5**^**−7**^**)**	**6 (0.001)**	2 (0.05)	**−3 (0.003)**	3 (0.1)	2 (0.1)	−0.2 (0.9)	−2 (0.1)	1 (0.3)		**5 (0.03)**	
PD	**23 (3**^**−9**^**)**	**6 (1**^**−4**^**)**	1 (0.6)	**−5 (1**^**− 4**^**)**	**7 (0.002)**	**2 (0.04)**	−1 (0.5)	**− 4 (0.001)**	1 (0.4)		**4 (0.04)**	
ACE	**17 (2**^**−7**^**)**	**6 (1**^**−4**^**)**	1 (0.6)	**−5 (1**^**− 4**^**)**	**9 (0.0003)**	**2 (0.05)**	−2 (0.2)	**−4 (0.001)**	1 (0.3)		2 (0.2)	
Simpson 1-D	**13 (7**^**−6**^**)**	**5 (1**^**−4**^**)**	−2 (0.2)	**4 (0.002)**	2 (0.1)	2 (0.3)	0.4 (0.9)	2 (0.1)	0.1 (0.8)		4 (0.1)	
Beta-diversity comparisons	Overall	EJ vs LJ	LJ vs MA	EJ vs MA	Overall	EJ vs LJ	LJ vs MA	EJ vs MA	EJ vs MA		EJ vs MA	
Unifrac Unweighted	**0.04 (9**^**−5**^**)**	**0.1 (0.001)**	**0.02 (0.01)**	**0.1 (0.001)**	**0.1 (9**^**−5**^**)**	**0.04 (0.001)**	**0.1 (0.001)**	**0.1 (0.001)**	**0.02 (9**^**−5**^**)**		**0.03 (9**^**−5**^**)**	
Unifrac Weighted	**0.1 (9**^**−5**^**)**	**0.1 (0.001)**	**0.1 (0.001)**	**0.1 (0.001)**	**0.1 (9**^**−5**^**)**	0.02 (0.1)	0.02 (0.2)	**0.04 (0.01)**	0.01 (0.3)		**0.04 (9**^**−5**^**)**	
Bray-Curtis	**0.1 (9**^**−5**^**)**	**0.1 (0.001)**	**0.1 (0.001)**	**0.03 (0.02)**	**0.1 (9**^**−5**^**)**	**0.1 (0.001)**	**0.1 (0.001)**	**0.1 (0.001)**	**0.02 (0.001)**		**0.03 (2**^**−4**^**)**	

Variation in alpha-diversity was assessed using Linear Mixed Effect models, with age groups as a fixed factor and sampling time as a random factor. Differences in beta-diversity were assessed using PERMANOVA. For each linear model effect test (alpha-diversity) we report the F statistic and significance (*P* value) and for each PERMANOVA test (beta-diversity) we report the R2 statistics and significance (*P* value). Significant differences are indicated in bold

*EJ* early juveniles, *LJ* late juveniles, *MA* mature adults, *J* juveniles

#### Beta-diversity

**Fig. 2:**
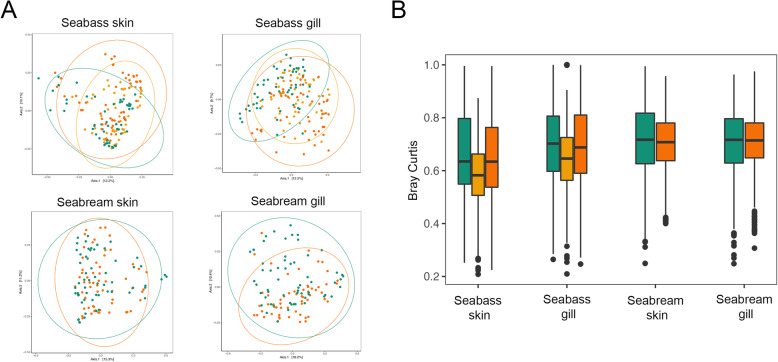
**a** PCoA plots computed using Bray-Curtis distances. Each dot represents a microbiome sample and is colored by age group (green: early juveniles/juveniles; yellow: late juveniles; orange: mature adults) in seabass *Dicentrarchus labrax* and seabream *Sparus aurata*. Ellipses denote a 95% confidence for the age group mean. **b** Bray-Curtis beta-diversity within age groups plotted for the early juveniles/juveniles (green), late juveniles (yellow) and mature adults (orange) of the seabass *Dicentrarchus labrax* and the seabream *Sparus aurata* (*n* = 60 per species x age group x tissue)

Microbial structure was estimated using phylogenetic UniFrac (unweighted and weighted) and Bray-Curtis distances. The PERMANOVA analyses of dissimilarity (diversity~age group, strata = sampling date) showed significant differences between age groups in both species (*p* < 0.02, Table 1), except for the UniFrac Weighted distance between the gills of early and late seabass juveniles (*p* = 0.1, Table 1), seabass late juveniles and mature adults (*p* = 0.2, Table 1), and the skin of juveniles and seabream adults (*p* = 0.3, Table 1). Moreover, high inter-individual variability within age groups was also observed. However, differences in beta-diversity dispersion within age groups for the skin and gill microbiotas of both species were small (Bray-Curtis distance, Fig. 2).

#### Bacterial taxa

**Fig. 3:**
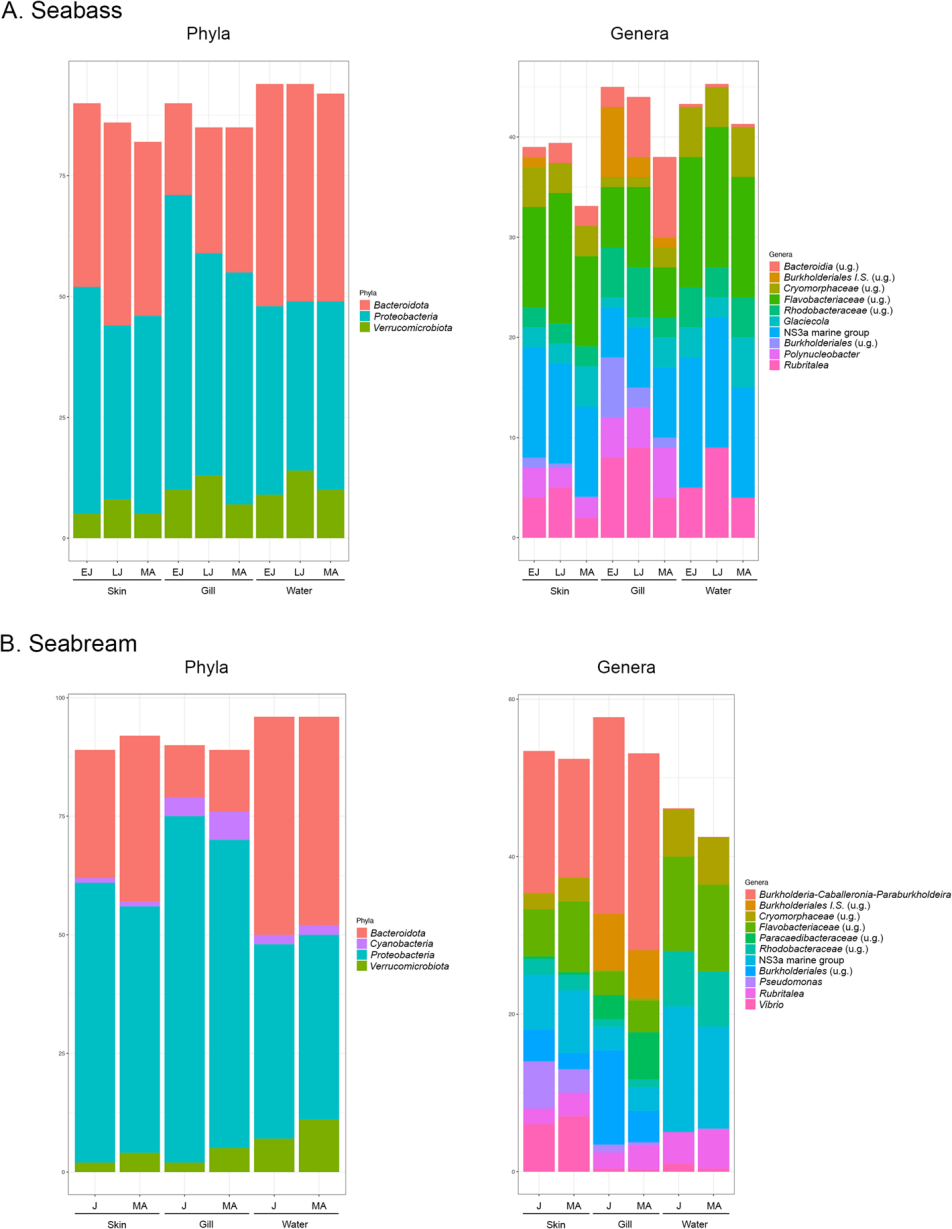
Most abundant (≥5%) phyla and genera of the seabass *Dicentrarchus labrax* (**a**) and the seabream *Sparus aurata* (**b**). Distinct bars represent relative abundance of each taxa for skin, gill and water microbiota of the studied age group (EJ: early juveniles, LJ: late juveniles, J: Juveniles, and MA: mature adults), labeled to the lowest taxonomic level possible (*n* = 60 per species x age group for tissues; *n* = 10 per species x age group for water). Unknown genera are identified as u.g

*Proteobacteria* and *Bacteroidetes* were the most abundant (≥5%) phyla in the skin (averaging 41 ± 4% and 39 ± 2% of the sequences in seabass and 55 ± 4% and 31 ± 4% in seabream) and gill (averaging 52 ± 7% and 25 ± 5% in seabass and 69 ± 4% and 12 ± 1% in seabream) microbiotas of all studied age groups (Additional file 4). The NS3a marine group and a genus belonging to the *Flavobacteriaceae* family were the most abundant (≥5%) genera in the skin (10 ± 1 and 11 ± 2, respectively) and gill (6 ± 1 for both) of all the age groups in seabass; while *Burkholderia-Caballeronia-Paraburkholderia* was the most abundant genus in the skin (17 ± 1) and gill (25 ± 0) of both age groups in seabream (Additional file 4). The most abundant microbial phyla and genera found in both fish species varied between age groups and tissues (Fig. 3, Additional file 4). LME models showed that the relative abundance of all those phyla was significantly different between age groups, except in the gill microbiota of the seabream, where the relative abundance of *Cyanobacteria* did not vary (Additional file 5). LME analyses also revealed that 100 and 63% of the genera varied between age groups in the skin and gill of the seabass, respectively, while 40 and 50% varied in the skin and gill of the seabream, respectively (Additional file 5). Pairwise comparisons of taxa across age groups in seabass yielded a higher percentage of significant differences between early juveniles and mature adults in both tissues (100% in the skin and 38% in the gill) than between early and late juveniles (67% in the skin and 13% in the gill), or between late juveniles and mature adults (0% in the skin and 25% in the gill) (Additional file 5).

### Microbial predicted functional diversity across age groups

**Fig. 4:**
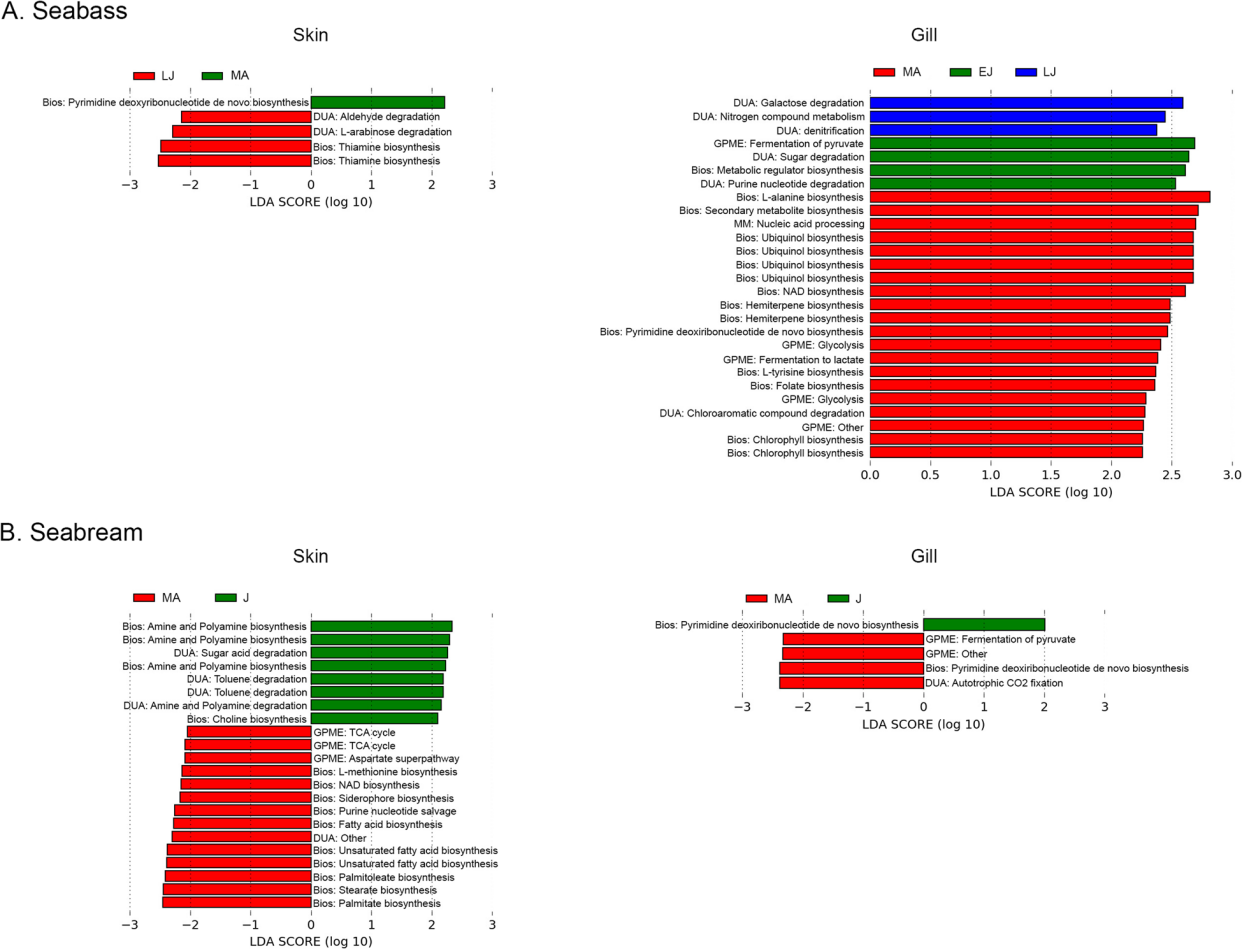
LDA score of differentially abundant enriched pathways in the skin and gill microbiota of early juveniles (EJ), late juveniles (LJ), juveniles (J) and mature adults (MA) of seabass *Dicentrarchus labrax* (**a**) and seabream *Sparus aurata* (**b**). Bios: Biosynthesis; DUA: Degradation/Utilization/Assimilation; GPME: Generator of Precursor Metabolites and Energy; MM: Macromolecule Modification

About 462 ± 18 KEGG pathways were inferred in the skin and gill microbiota of the seabass, while 455 ± 4 pathways were inferred in the skin and gill microbiota of the seabream. Linear discriminant analysis of the metagenomic predictions performed in LEfSe showed that different pathways were significantly enriched for each age group in both species (Fig. 4, Additional file 6). Overall, there were more enriched pathways in the older age groups (seabass late juveniles and mature adults of both species) than in the younger age groups of both species (Fig. 4, Additional file 6).

While there were no significantly enriched pathways in the skin microbiota of early juvenile seabass, enriched pathways in the gill microbiota of this age group were related to metabolic regulator biosynthesis, purine nucleotide degradation, sugar degradation and fermentation of pyruvate. In the skin microbiota of late juvenile seabass, enriched pathways were related to thiamine biosynthesis, aldehyde degradation and L-arabinose degradation; while in the gill microbiota they were related to denitrification, galactose degradation and nitrogen compound metabolism. In mature seabass, pyrimidine and purine deoxyribonucleotide de novo biosynthesis were enriched in both tissues. Additionally, the gill microbiota was also enriched by pathways related to the biosynthesis of chlorophyll, folate, hemiterpene, L-alanine, L-tyrosine, NAD, secondary metabolite and ubiquinol, chloroaromatic compound degradation, fermentation to lactate and glycolysis (Fig. 4, Additional file 6).

In the skin microbiota of seabream juveniles, enriched pathways were related to amine and polyamine biosynthesis and degradation, choline biosynthesis, and sugar acid and toluene degradation; whereas in the gill microbiota only pyrimidine and purine deoxyribonucleotide de novo biosynthesis were identified. The enriched pathways of the seabream mature adults were related to fatty acid, L-methionine, NAD, palmitate, palmitoleate, siderophore, stearate and unsaturated fatty acid biosynthesis, pyrimidine and purine nucleotide salvage, aspartate superpathway and TCA cycle in the skin microbiota; whereas pyrimidine and purine deoxyribonucleotide de novo biosynthesis, autotrophic CO_2_ fixation and fermentation of pyruvate were enriched in the gill microbiota (Fig. 4, Additional file 6).

### Fish and water microbiota comparisons

**Fig. 5:**
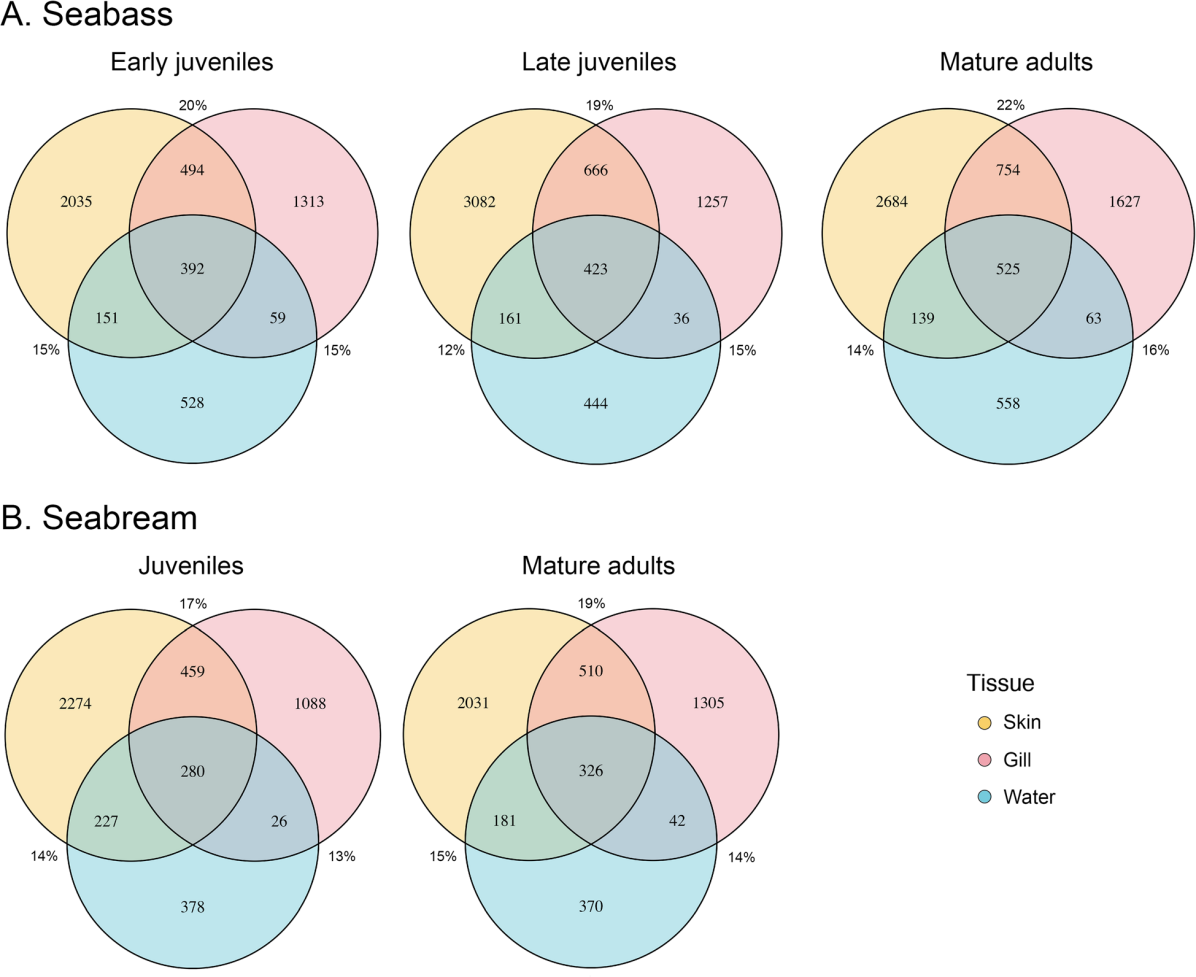
Venn diagrams showing the number and percentage of shared ASVs between skin (yellow), gill (pink) and water (blue) microbiota of the different age groups for the seabass *Dicentrarchus labrax* (**a**) and the seabream *Sparus aurata* (**b**) (*n* = 60 per species x age group for tissues; *n* = 10 per species x age group for water)

The microbiota of the farm fishpond waters showed higher alpha-diversity than the skin and gill microbiota of seabass and seabream for all the indices, except for the Shannon index in the seabass late juveniles (Additional file 2). The analyses of dissimilarity between the skin and gill microbiotas and the water microbiota were statistically significant for all pairwise comparisons (PERMANOVA, *p* < 0.001, Table 2). Moreover, results from the Mantel tests revealed a correlation between gill and water microbiota of seabass and seabream across age groups (*p* < 0.03, Table 2), except in the case of late juvenile seabass (*p* > 0.05, Table 2). PCoAs showed that the water microbiota clustered more closely to the skin microbiota than to the gill microbiota in both fishes (Additional file 7). In both species, the percentage of ASVs shared between skin and water microbiota, and between gill and water microbiota was very similar (14% ± 1 and 15% ± 1 of ASVs, respectively) (Fig. 5).

**Table 2:** Results from pairwise comparisons of beta-diversity and Mantel tests for fish tissues and water per age group for the seabass *Dicentrarchus labrax* and the seabream *Sparus aurata* (*n* = 60 per species x age group for tissues; *n* = 10 per species x age group for water)

			UniFrac Unweighted		UniFrac Weighted		Bray-Curtis	
			Permanova	Mantel	Permanova	Mantel	Permanova	Mantel
Seabass	EJ	Skin vs Water	**0.1 (0.001)**	**0.2 (0.04)**	**0.1 (0.01)**	−0.1 (0.9)	**0.04 (0.04)**	0.1 (0.2)
		Gill vs Water	**0.2 (0.001)**	**0.5 (1**^**−4**^**)**	**0.1 (0.001)**	**0.4 (1**^**−4**^**)**	**0.2 (0.001)**	**0.4 (1**^**−4**^**)**
	LJ	Skin vs Water	**0.1 (0.001)**	**0.2 (0.01)**	**0.1 (0.001)**	−0.04 (0.7)	**0.1 (0.001)**	0.1 (0.1)
		Gill vs Water	**0.2 (0.001)**	**0.5 (1**^**−4**^**)**	**0.2 (0.001)**	−0.2 (0.9)	**0.2 (0.001)**	**0.4 (1**^**−4**^**)**
	MA	Skin vs Water	**0.1 (0.001)**	0.2 (0.1)	**0.1 (0.001)**	−0.1 (0.8)	**0.1 (0.002)**	0.1 (0.1)
		Gill vs Water	**0.1 (0.001)**	**0.4 (2**^**−4**^**)**	**0.1 (0.001)**	**0.2 (0.03)**	**0.1 (0.001)**	**0.2 (0.01)**
Seabream	J	Skin vs Water	**0.1 (0.001)**	**0.2 (0.02)**	**0.04 (0.01)**	−0.1 (0.9)	**0.1 (0.001)**	0.1 (0.1)
		Gill vs Water	**0.2 (0.001)**	**0.5 (1**^**−4**^**)**	**0.1 (0.001)**	**0.6 (1**^**− 4**^**)**	**0.4 (0.001)**	**0.6 (1**^**− 4**^**)**
	MA	Skin vs Water	**0.1 (0.001)**	**0.2 (0.02)**	**0.03 (0.03)**	0.04 (0.3)	**0.2 (0.001)**	**0.2 (0.02)**
		Gill vs Water	**0.1 (0.001)**	**0.6 (1**^**−4**^**)**	**0.5 (0.001)**	**0.7 (1**^**−4**^**)**	**0.3 (0.001)**	**0.5 (1**^**−4**^**)**

For each PERMANOVA test we report the R2 statistics and significance (*P* value) and for each Mantel test we report the R statistic and significance (*P* value). Significant differences/associations are indicated in bold

*EJ* early juveniles, *LJ* late juveniles, *MA* mature adults, *J* juveniles

## Discussion

We characterized the skin and gill microbiota of different age groups of farmed European seabass and gilthead seabream using 16S rRNA amplicon high-throughput sequencing. By taking into account potential environmental and seasonal effects, our study shows that fish age, in particular sexual maturation and growth, impact skin and gill microbial diversity (Table 1; Fig. 1), composition (Additional file 4; Figs. 3, 5) and predicted microbial functions (Additional file 6; Fig. 4).

### Microbial diversity across age groups

Fish growth and sexual maturation are usually accompanied by extreme morphological and physiological changes (e.g., [44, 45]). Importantly, some of these changes reported for the skin and gills have been suggested to also affect their microbiota. For example, changes in epidermal structure derived from sexual maturation (e.g., increases in the number, size and activity of the mucous cells) have been detected in several fish species (e.g., [44, 46]), and suggested to increase infection rate with *Saprolegnia* fungus in sea trout and brown trout [47]. Likewise, changes in the hormones expressed in the skin alter the biochemistry of the skin mucous and also potentially affect its microbiota [48]. Fish growth and sexual maturation also impact gill morphology and function in some fish species. For example, the ability to osmoregulate at different salinities was seen to increase throughout the developmental stages (larva to juvenile) of seabass [45]. Additionally, body size was also identified as the main factor affecting morphological variation in gill rakes and gill pore size in the Silver Carp and Gizzard Shad, suggesting that the overall filtering ability of these species is related to size and maturation [49]. Importantly, a recent study showed that body weight increase is accompanied by higher microbial community structuring in the skin and gill of rabbit fish [36]. We thus hypothesize that such physiological and morphological changes occurring during fish growth have led to the changes in microbial diversity, composition and predicted functionality observed in the present study.

The seabass skin and gill microbiotas of older age groups showed significantly higher alpha-diversity than those of early juveniles. Additionally, a higher percentage of significant differences in the relative abundance of the most abundant phyla and genera occurred between early juveniles and older age groups (67 ± 27% and 55 ± 38% in the skin and gill, respectively; Additional file 5). This suggests that the skin and gill microbiotas of the seabass were highly dynamic, diversifying with age. Conversely, the skin and gill microbiotas of the seabream juveniles and adults showed similar alpha-diversity means, although a high percentage of the most abundant phyla and genera varied between age groups (70 ± 42% and 63 ± 18% in the skin and gill, respectively; Additional file 5). Variation in microbiota alpha-diversity between different age groups has been previously reported for many fish species. For example, studies on the zebrafish and salmon gut microbiota, have reported differences between mature and immature life stages; however, those differences also coincide with other major ecological changes in the fish, such as diet [17] or environment transitions [20]. Moreover, the relative abundance of predominant bacterial groups also changed with aging in other fish species (e.g., [14, 17, 20]).

We detected significant differences in microbial structure across all age groups in both species. Similar results have been also reported in other fish (e.g., several reef fish species [14, 35] and *Salmo salar* [19]), being particularly evident in longitudinal studies encompassing several months [13, 17, 20]. High inter-individual variability within age groups was also previously reported for other fish species (e.g., [14, 28, 30]). However, our results showed that fish age only explained a low percentage of the variation in the bacterial community structure (R2 < 0.1). This suggests that microbial differences between age groups are small at the community level, but clearly noticeable at the species level, with a high proportion of the predominant bacterial taxa (61 ± 39% and 46 ± 32% in the skin and gill of seabass, respectively; 70 ± 42% and 63 ± 18% in the skin and gill of the seabream, respectively) changing their abundance with sexual maturation. Although our statistical models accounted for sampling dates as a random factor, other biotic and abiotic factors (e.g., variation in the environment and individual weights) could be responsible for most of the variation observed in community structure (e.g., [12, 19, 28]).

### Microbial predicted functional diversity across age groups

The predicted functional analysis suggests that distinct significantly enriched metabolic pathways are expressed in skin and gill microbiotas of both fish species across age groups. Although metabolic information is particularly limited for fish microbiotas, studies on other vertebrates, mainly in humans and their gut microbiotas, are starting to shed light on the beneficial outcomes specific microbial metabolic functions have on the host health and physiology (e.g. [50]).

Notwithstanding that present results should be interpreted with caution since PICRUSt2 analysis is limited by the currently available genomes and biased towards human health microorganisms [51], one could suggest that some of the enriched metabolic pathways found in our analysis could also improve the seabass and seabream health and physiology. The protective role of the microbiota is often related to the production of secondary metabolites that provide chemical defense and mediate bacterial diversity [4]. Secondary metabolites with antimicrobial activity have been previously isolated from microbial species inhabiting the gut microbiome of fish [52]. Here, the biosynthesis of secondary metabolites that have been associated with antimicrobial activity, including hemiterpene (e.g., [53]), were enriched in mature adults of both species. Additionally, the biosynthesis of chlorophyll and several amino acids, herein enriched in older age groups of both fish species, have also been found to be expressed in the skin and gut of healthy humans (e.g., [54, 55]). Importantly, amino acid biosynthesis was reported in the gut microbiota of grass carp when fed a protein-deficient diet, suggesting a metabolic role of the gut microbiota towards fish nutrition [56]. The biosynthesis of vitamins, here enriched in older age groups of seabass, has been found beneficial for human skin (e.g., [57]) and gut mucosa, including folate and thiamine [58]. In addition, polyamines are bacterial metabolites known to have several benefits towards gut mucosa recovery [59]. These pathways were also enriched in the juveniles of seabream.

It is also worth noticing that some of the enriched metabolic pathways detected in the present study could be driven by the high environmental variability of the Alvor estuary where these fish were reared. In estuaries, salinity variations occur on a daily basis due to tides and pollutants can be prevalent (e.g., [60]). Biosynthesis of fatty acids and unsaturated fatty acids were two of the predicted metabolic pathways enriched in the microbiota of mature seabream. These same pathways have also been enriched in previous analyses of the skin and gut microbiota in the atlantic salmon [61, 62] and in the skin microbiota of the common snook [63] when transitioning between freshwater and seawater. Additionally, two of the predicted metabolic pathways identified in both fish species were related to degradation of toxic compounds. Specifically, biodegradation of the highly prevalent toxic pollutants toluene and chloroaromatic compounds by bacteria is essential to remove them from the environment and to prevent absorption through the skin and gills in aquatic animals [64–66].

Following alpha-diversity patterns, fish from older age groups, particularly in the seabass, had greater enrichment of predicted functions related mainly to the biosynthesis and degradation of compounds; as well as, to a lesser extent, metabolism and energy cycles. We then hypothesize that the increase in microbial diversity observed as fish ages leads to wider functional diversity. This could prove beneficial to those fishes, given the key physiological modifications older fish groups are experiencing during sexual maturation and growth.

### Fish and water microbiota comparisons

The water microbiotas of fishponds were significantly distinct and more diverse than the skin and gill microbiota of both fish species, regardless of their age. It is known that free-living microbial communities retain higher richness than host-associated communities [31], with many studies showing a higher bacterial diversity in water relative to fish skin [28, 30, 36, 67–69], gills [14, 36], gut [7, 15, 18, 21, 70], stomach [36], hindgut [36] and whole larvae [22]. Although some studies in fish have shown that the microbial communities found in the water tend to be recovered in the larval gut microbiota [17, 21], others have also shown that water microbiota does not influence directly the microbiota of the fish mucosa [7, 8, 13–15, 18, 19, 22, 28, 30, 34, 36, 67–69, 71, 72]. Importantly, a previous study of the skin microbiotas of seabass and seabream also showed significant differences with planktonic communities [68]. However, in that study only a low number of Operational Taxonomic Units (3%) was shared between skin and water microbiota; whereas in the present study higher percentages of ASVs were shared between the skin (14% ± 1) and the gill (15% ± 1) of both fish species and the surrounding water.

Microbial dissimilarities depicted by PCoAs showed that, although significantly different, the skin microbiota of both species clustered more closely to the water microbiota than the gill microbiota. However, only a small percentage of the variation (PC 1 – average 18% ± 2; PC 2 – average 10% ± 1) was explained by this analysis. On the other hand, the results from the Mantel tests showed a significant (*p* < 0.03) correlation between the water and gill microbiotas, but not between the water and skin microbiotas. This suggests that although both skin and gill are permanently in contact with water, the gill environment may be more susceptible to variations in the water microbiota.

## Conclusions

Skin and gill are important mucosal barriers that protect the fish from the external environment. They are in permanent contact with the water column and thus prone to pathogenic bacterioplankton colonization. However, most studies so far investigating microbial changes related to fish age have either strictly focused on early life stages (i.e., larvae development) or on the gut microbiota. In the present study, we demonstrate that, to some extent, changes occurring later in life can also be correlated with aging factors such as growth and sexual maturation. We uncovered important differences in the diversity, composition and predicted functionality of the skin and gill microbiotas across age groups of farmed seabass and seabream. Fish included in this study were exposed to a heterogeneous estuarine environment, that varies seasonally as well as daily (e.g., salinity and temperature fluctuations). Hence, although we observed significant differences in microbial community structure due to age, other biotic and abiotic factors not considered here may have more deeply structured the fish microbiota. Growth rate decreased drastically with age, being much higher in juveniles (243 and 83% for early and late seabass juveniles, and 143% in seabream) relative to adults (43 and 16% in adult seabass and seabream, respectively). We, thus, conclude that growth and sexual maturation are likely the main drivers of the microbial differences attributed to age observed here. Overall, our results agree well with other studies of the skin [35, 36] and gill [14, 36] microbiotas of several wild reef fish, suggesting this could be a general pattern across fish. Our results also highlight the importance of aging in farm fish studies focused on microbial dysbiosis and disease dynamics.

## Material and methods

### Fish species, sampling and preparation

Fish were sampled at a semi-intensive open-water farm in the Alvor Estuary (Ria Formosa, Portimão, Portugal). In this fish farm, seabass and seabream production can take up to 36 months to reach minimum commercial size, so having a healthy mucosa during this time is of utter importance. The gilthead seabream is a protandric hermaphrodite, maturing first as males between years 1 and 2 in the wild, with sex reversal occurring in the following 2–3 years [38–40]. The European seabass reaches sexual maturity between years 2 and 3 in males, and after year 3 in females [41–43]. In this particular fish farm, seabass typically reaches sexual maturity at approximately 275 g, whereas for seabream maturity is usually attained at 300 g. We monitored the skin and gill microbiota of seabass and seabream of different age cohorts, including juveniles and adults. Due to commercial restrictions within the fish farm, sampling was strictly non-invasive and fish could not be dissected to confirm sexual maturation. The categorization of the age group cohorts was based on previous studies (e.g. [38, 41]) and the weight at maturity records available at the farm.

We collected samples every other week (12 sampling time points) between August 2017 and January 2018 (6 months). We simultaneously sampled three seabass age groups with approximately 1 year difference. Seabass specimens were categorized as early juveniles (9 months and an average weight of 22 g at the beginning of the study and 15 months and an average weight of 76 g at the last sampling point), late juveniles (18 months and an average weight of 151 g at the beginning of the study and 24 months and an average weight of 277 g at the last sampling point), and mature adults (32 months and an average weight of 467 g at the beginning of the study and 38 months and an average weight of 669 g at the last sampling point). We also simultaneously sampled two seabream cohorts categorized as juveniles (15 months and an average weight of 103 g initially and 21 months and an average weight of 250 g at the last sampling point), and mature adults (37 months and an average weight of 411 g at the beginning of the study and 37 months and an average weight of 476 g at the last sampling point). Seabream from an intermediate age-cohort were not available.

Each age group and species was reared in separate but not distant open-water ponds (maximum 344 m and 380 m apart for seabass and seabream, respectively; Additional file 1). In this fish farm, all ponds share the same water inflow, which is taken from a single point in the estuary. Water in each pond is naturally recycled at each high tide (twice a day) and never shared between ponds. Hence, fish share roughly the same water quality and environment. Additionally, fry were bought from commercial hatcheries, which genetic variation is considered low [73].

Fish were caught from each tank using a fishing line, and skin and gill samples were non-invasively taken using sterile swabs (Medical Wire & Equipment, UK). We swabbed the right filaments between the first and second arches of the gill and the right upper lateral part of the fish skin from head to tail. Afterwards fish were released unharmed. We collected water samples (1 l) from the five different culture ponds at the same time as fish swabbing was performed, except during the month of December, when no water samples could be collected. Water samples were filtered through 0.2 μm cellulose nitrate filters on collection day. Swabs and filters were immediately frozen at − 20 °C and then transported in dry ice to the CIBIO-InBIO laboratory where they were kept at − 80 °C until processing.

We sampled five fish per week per age group, totaling 60 specimens per species and age group. We processed 360 seabass samples (60 skin and 60 gills × 3 age groups) plus 29 water samples from their corresponding fishponds. We also processed 240 seabream samples (60 skin and 60 gills × 2 age groups) plus 16 water samples from their corresponding fishponds. Seabass and their corresponding water samples were processed using the PowerSoil DNA Isolation Kit (QIAGEN, Netherlands), while seabream and corresponding water samples were processed using the PureLink Microbiome DNA Purification Kit (ThermoFisher Scientific, UK). We used two different DNA extraction kits due to supply shortage at the time of extraction. This technical difference did not impact the goals of our study since we studied each fish species separately (i.e., mucosa microbiotas are not compared between fish species). We measured DNA concentration and quality in a NanoDropTM 2000 Spectrophotometer (ThermoFisher Scientific, USA). DNA extractions were shipped in dry ice to the University of Michigan Medical School (USA) for amplification and sequencing according to the protocol of Kozich et al. [74]. Each sample was amplified for the V4 hyper-variable region of the 16S rRNA gene (~ 250 bp). All amplicon libraries were pooled and sequenced in a single run of the Illumina MiSeq sequencing platform.

Approximately 8,313,608 and 6,943,265 16S rRNA sequences were retrieved for seabass and seabream, respectively. The number of sequences per sample ranged from 726 to 46,001 in seabass and from 5145 to 151,713 in seabream. After normalization and removal of non-bacterial reads, 8724 and 5754 ASVs were assigned to the skin and gill, respectively, of seabass; while 5308 and 3423 ASVs were assigned to the skin and gill, respectively, of seabream. A total of 2543 ASVs were retrieved from the water samples collected in seabass fishponds, while 1440 ASVs were retrieved from the waters of seabream fishponds. Microbial taxa showing a mean relative proportion ≥ 5% in any group were considered the most abundant taxa in that group.

### Data processing and statistical analysis

Raw FASTQ files were denoised using the DADA2 pipeline in R with the parameters for filtering and trimming being trimLeft = 20, truncLen = c (220,200), maxN = 0, maxEE = c (2,2), truncQ = 2 [75]. We estimated a midpoint rooted tree of ASVs using the Quantitative Insights Into Microbial Ecology 2 package (QIIME2; release 2019.7). We constructed a table containing amplicon sequence variants (ASVs) and made taxonomic inferences against the SILVA (138 release) reference database [76]. We normalized ASV abundances using the negative binomial distribution [77], which accounts for library size differences and biological variability.

Microbial taxonomic alpha-diversity (intra-sample) was calculated using Shannon, Faith’s phylogenetic diversity (PD), ACE and Simpson 1-D indices as implemented in the R package phyloseq [78]. We assessed variation in microbial composition (alpha-diversity) and the mean proportions of the most abundant taxa (≥5% of all sequences) using Linear Mixed Effects models (LME) with the lmer R package [79]. Since we were interested in assessing whether microbial diversity varied across fish age groups (predictor), we used age groups as a fixed factor and sampling date (with 12 sampling time points) as a random factor. The final general LME formula was expressed as: microbial diversity~fish age group + (1|sampling time point). Microbial structure (beta-diversity) was estimated using phylogenetic Unifrac (unweighted and weighted) and Bray-Curtis distances. Dissimilarity in microbial structure between samples was visualized using principal coordinates analysis (PCoA). Additionally, differences in community structure driven by fish age group were further tested using permutational multivariate analysis of variance (PERMANOVA) as implemented in the adonis function of the vegan R package [80]. We used the strata argument to permutate sampling dates and ran 1000 permutations.

Previous fish studies of skin and gill microbiota (e.g., [7, 8, 36, 70]), including seabass and seabream [81], have shown remarkable differences in microbial composition and structure across host species and tissues. Although the two species studied here are farmed in the same location, provided feeds were different throughout the sampling period. For this reason, and because fish samples were processed using two different DNA extraction kits, we did not compare mucosa microbiotas between fishes (e.g., [21, 82]). Additionally, a previous study by our group [83] showed that disease and antibiotic treatment in seabass leads to asymmetrical shifts in skin and gill microbial communities. Therefore, we carried out all our statistical analyses separately for each fish species and tissue.

Microbial potential metabolic functions were predicted using the metagenomic Phylogenetic Investigation of Communities by Reconstruction of Unobserved States software (PICRUSt2) embedded in QIIME2 [84], applying a weighted nearest sequenced taxon index (NSTI) cutoff of 0.03. Predicted metagenomes were collapsed using the Kyoto Encyclopedia of Genes and Genomes (KEGG) Pathway metadata [85]. We identified differentially abundant metabolic pathways in the skin and gill microbiota of seabass and seabream across age groups using linear discriminant analysis (LDA) in LEfSe, using age groups as classes [86]. As suggested by the authors, we used a *P*-value cut-off of 0.05 and a LDA effect size cut-off of 2 [86].

Finally, to assess to what extent water microbial communities shaped skin and gill microbiota across fish age groups, we estimated the number of shared ASVs between fish and water microbiota and constructed Venn diagrams in R. We used PERMANOVA and mantel testes [87] to assess differences in community structure and correlations between tissues and water microbiota, respectively, in both species.

## Supplementary Information

**Additional file 1.** Illustrative scheme of the semi-intensive fish farm where samples were collected. All ponds shared the same inflow of estuarine water (A) and water was never shared between ponds. Each age group and species was reared in separated but not distant open water ponds: 1 - mature adults seabass; 2 - mature adults seabream; 3 - late juveniles seabass; 4 - early juveniles seabass; 5 - early juveniles seabream.

**Additional file 2 **Mean values and standard deviations of Shannon, Faith’s phylogenetic (PD), ACE and Fisher alpha-diversity estimates plotted for skin (yellow), gill (pink) and water (blue) microbiota of the different age groups of the seabass *Dicentrarchus labrax* (A) and the seabream *Sparus aurata* (B) (*n* = 60 per species x age group for tissues; *n* = 10 per species x age group for water).

**Additional file 3 **Mean values and standard deviations of Faith’s phylogenetic (PD), ACE and Fisher alpha-diversity estimates plotted for the early juveniles/juveniles (green), late juveniles (yellow) and mature adults (orange) of the seabass *Dicentrarchus labrax* (A) and seabream *Sparus aurata* (B) (*n* = 60 per species x age group x tissue). Pairwise comparisons of alpha-diversity were assessed using Linear Mixed Effect models with age groups as a fixed factor and sampling date as a random factor. Statistically significant differences are denoted with an asterisk and non statistically significant differences are denoted with “ns”.

**Additional file 4.**

**Additional file 5.**

**Additional file 6.**

**Additional file 7 **PCoA plot computed using Bray-Curtis distances for water, skin and gills microbiota of the seabass *Dicentrarchus labrax* (A) and the seabream *Sparus aurata* (B) (*n* = 60 per species x age group for tissues; *n* = 10 per species x age group for water). Each dot represents a microbiome sample and is coloured by tissue/origin (skin, gill and water).

## Data Availability

The datasets generated and/or analyzed during the current study are available in the NCBI Sequence Read Archive (SRA) database within the BioProject ID PRJNA687505.

## Abbreviations

ACE: Abundance-based Coverage Estimator
ASV: Amplicon Sequence Variant
CO_2_: Carbon dioxide
DADA: Divisive Amplicon Denoising Algorithm
DNA: Deoxyribonucleic Acid
KEGG: Kyoto Encyclopedia of Genes and Genomes
LDA: Linear Discriminant Analysis
LEfSe: Linear discriminant analysis Effect Size
LME: Linear Mixed Effects
NAD: Nicotinamide-Adenine Dinucleotide
NSTI: Nearest Sequenced Taxon Index
OTU: Operational Taxonomic Unit
PCoA: Principal Component Analysis
PD: Phylogenetic Diversity
PERMANOVA: Permutational Analysis of Variance
PICRUST: Phylogenetic Investigation of Communities by Reconstruction of Unobserved States
QIIME: Quantitative Insights Into Microbial Ecology
rRNA: Ribosomal Ribonucleic Acid
TCA: Tricarboxylic Acid Cycle
