# Supplementary figures and images for: Effects of aging on the skin and gill microbiota of farmed seabass and seabream
Source: Anim Microbiome. 2021 Jan 12;3:10. doi: 10.1186/s42523-020-00072-2 (PMC7934244; doi:10.1186/s42523-020-00072-2)

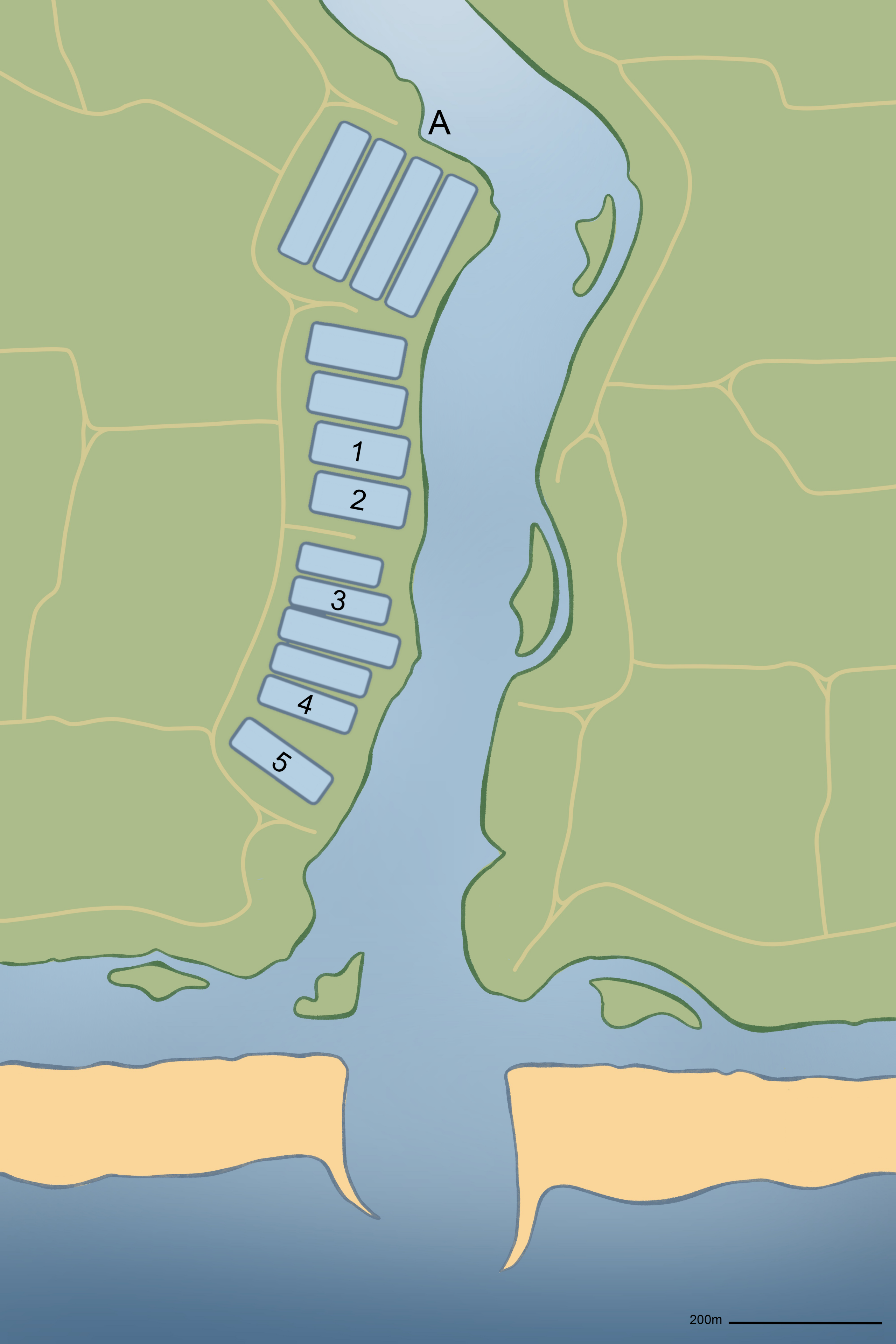

A

1

2

3

4

5

200m

Supplement: Supplementary file 1 — Additional file 1. Illustrative scheme of the semi-intensive fish farm where samples were collected. All ponds shared the same inflow of estuarine water (A) and water was never shared between ponds. Each age group and species was reared in separated but not distant open water ponds: 1 - mature adults seabass; 2 - mature adults seabream; 3 - late juveniles seabass; 4 - early juveniles seabass; 5 - early juveniles seabream. [file 42523_2020_72_MOESM1_ESM.pdf]

A. Seabass

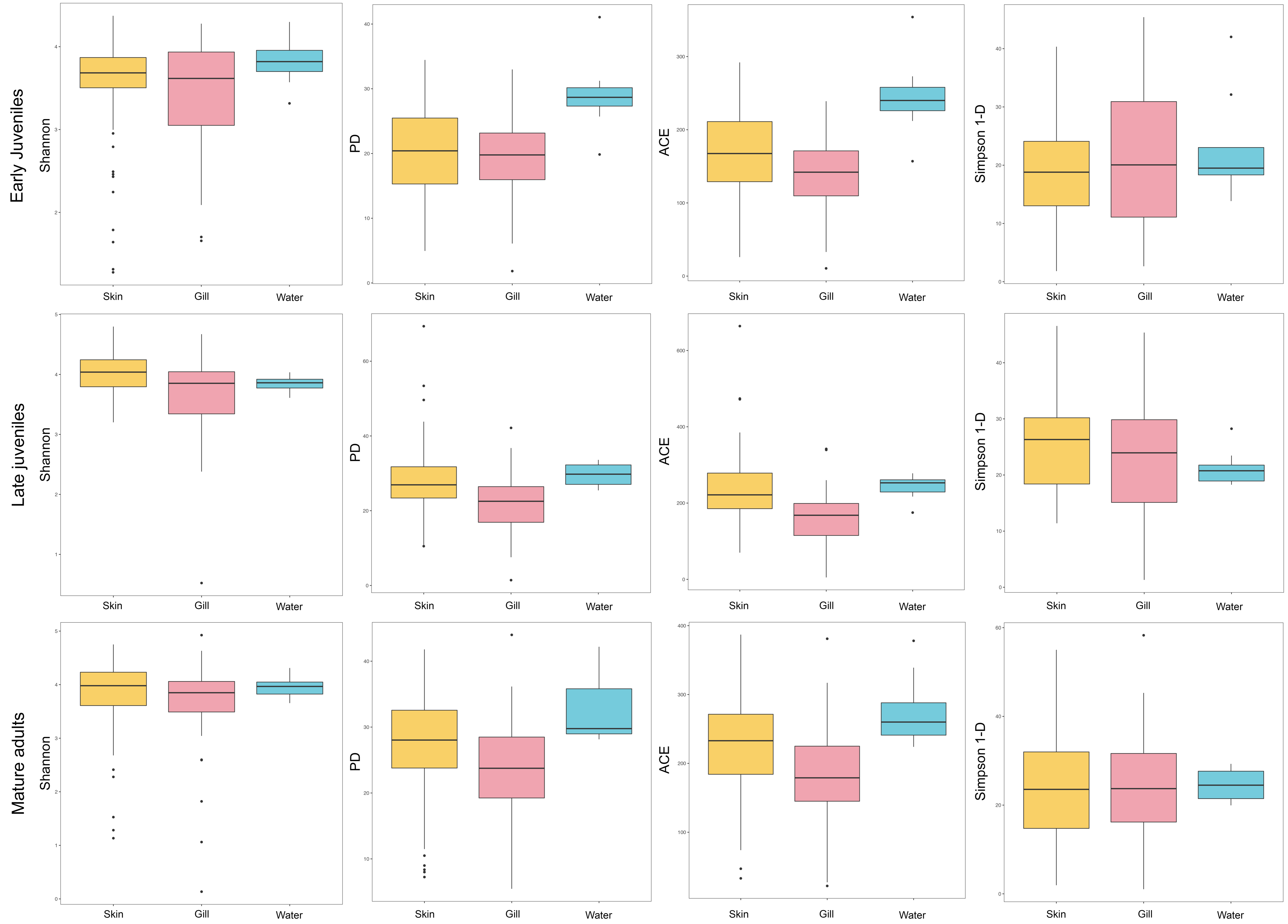

B. Seabream

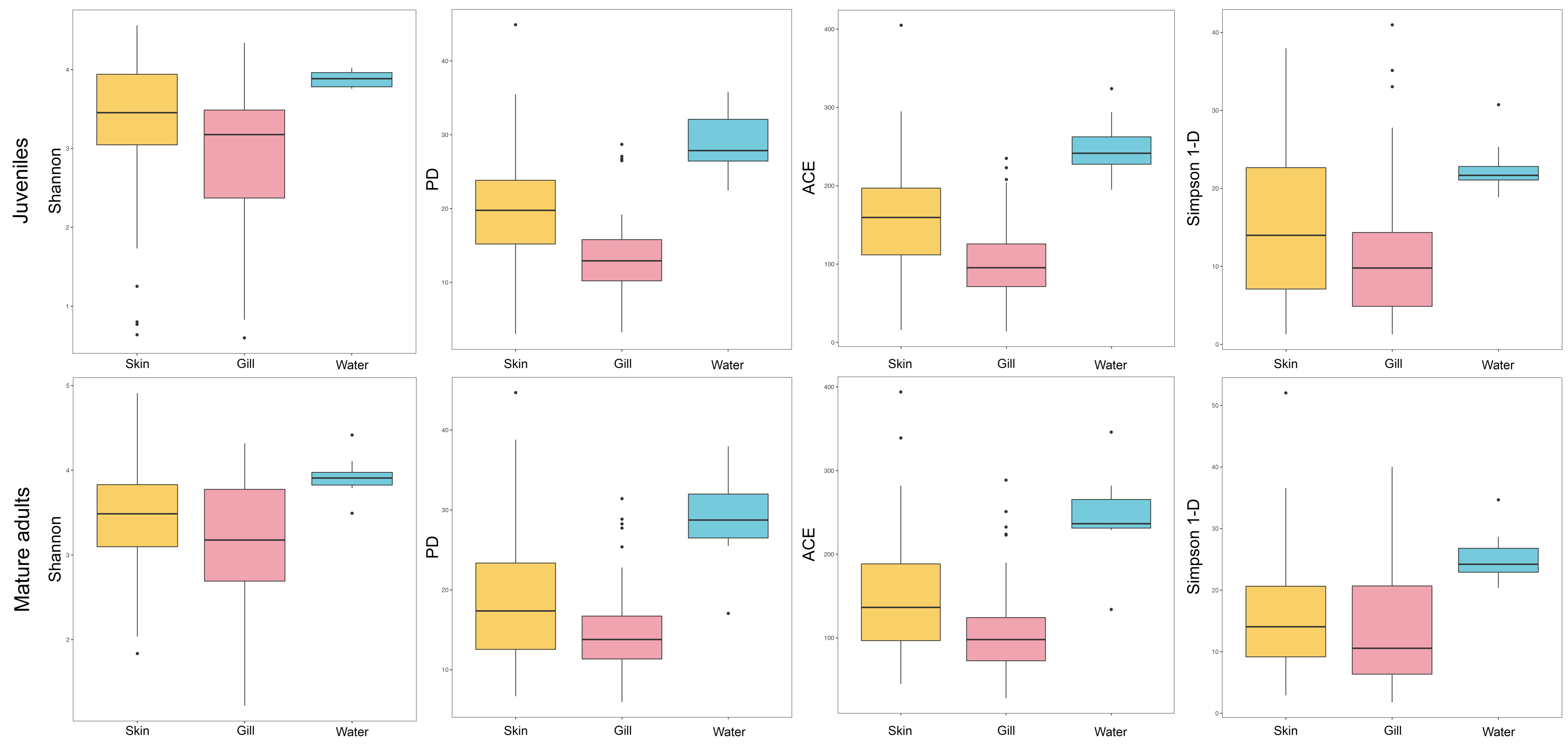

Supplement: Supplementary file 2 — Additional file 2 Mean values and standard deviations of Shannon, Faith’s phylogenetic (PD), ACE and Fisher alpha-diversity estimates plotted for skin (yellow), gill (pink) and water (blue) microbiota of the different age groups of the seabass Dicentrarchus labrax (A) and the seabream Sparus aurata (B) (n = 60 per species x age group for tissues; n = 10 per species x age group for water). [file 42523_2020_72_MOESM2_ESM.pdf]

A. Seabass

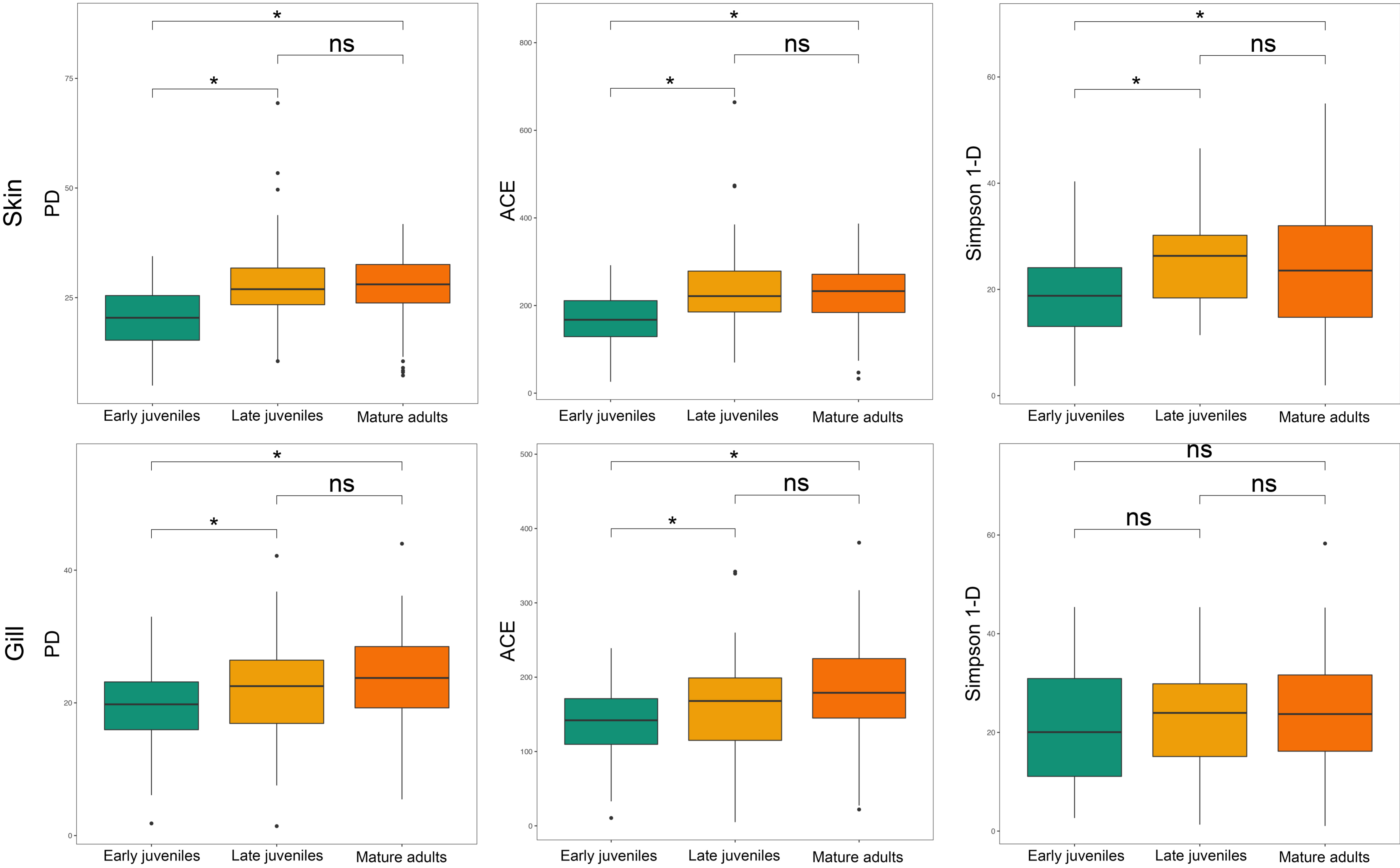

B. Seabream

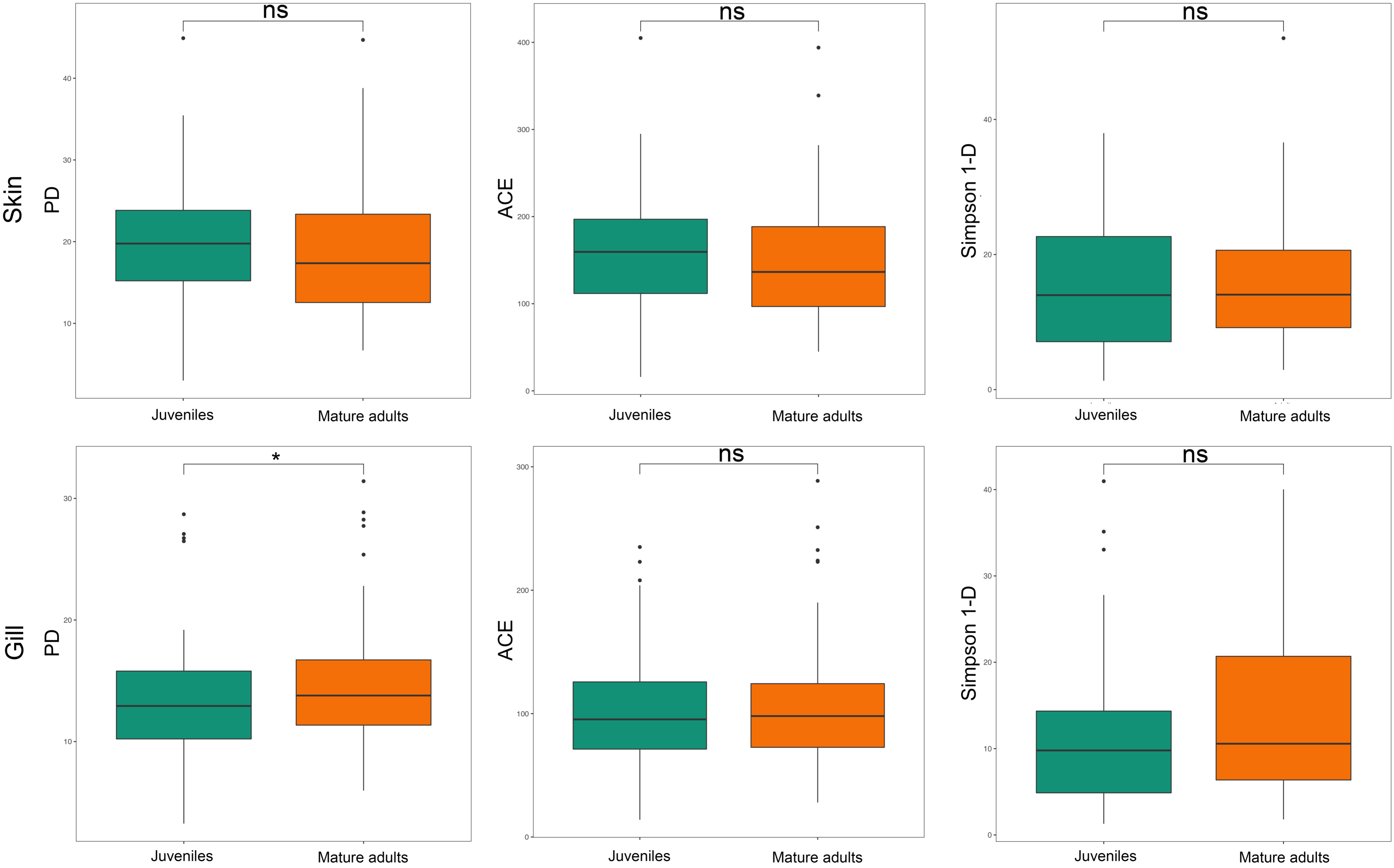

Supplement: Supplementary file 3 — Additional file 3 Mean values and standard deviations of Faith’s phylogenetic (PD), ACE and Fisher alpha-diversity estimates plotted for the early juveniles/juveniles (green), late juveniles (yellow) and mature adults (orange) of the seabass Dicentrarchus labrax (A) and seabream Sparus aurata (B) (n = 60 per species x age group x tissue). Pairwise comparisons of alpha-diversity were assessed using Linear Mixed Effect models with age groups as a fixed factor and sampling date as a random factor. Statistically significant differences are denoted with an asterisk and non statistically significant differences are denoted with “ns”. [file 42523_2020_72_MOESM3_ESM.pdf]
