# Supplementary material for: Effects of aging on the skin and gill microbiota of farmed seabass and seabream
Source: Anim Microbiome. 2021 Jan 12;3:10. doi: 10.1186/s42523-020-00072-2 (PMC7934244; doi:10.1186/s42523-020-00072-2)
Supplement: Supplementary file 4 — Additional file 4. [file 42523_2020_72_MOESM4_ESM.docx]

|  | Seabass | | | | | | | | |  | Seabream | | | | | |
| --- | --- | --- | --- | --- | --- | --- | --- | --- | --- | --- | --- | --- | --- | --- | --- | --- |
|  | Skin | | | Gill | | | Water | | |  | Skin | | Gill | | Water | |
|  | EJ | LJ | MA | EJ | LJ | MA | EJ | LJ | MA |  | J | MA | J | MA | J | MA |
| Phyla |  |  |  |  |  |  |  |  |  |  |  |  |  |  |  |  |
| *Bacteroidota* | **38** | **42** | **36** | **19** | **26** | **30** | **46** | **45** | **43** |  | **27** | **35** | **11** | **13** | **46** | **44** |
| *Cyanobacteria* | - | - | - | - | - | - | - | - | - |  | 1 | 1 | 4 | **6** | 2 | 2 |
| *Proteobacteria* | **47** | **36** | **41** | **61** | **46** | **48** | **39** | **35** | **39** |  | **59** | **52** | **73** | **65** | **41** | **39** |
| *Verrucomicrobiota* | **5** | **8** | **5** | **10** | **13** | **7** | **9** | **14** | **10** |  | 2 | 4 | 2 | **5** | **7** | **11** |
| Genera |  |  |  |  |  |  |  |  |  |  |  |  |  |  |  |  |
| *Burkholderia-Caballeronia-Paraburkholderia* | - | - | **-** | - | - | - | - | - | - |  | **18** | **15** | **25** | **25** | 0.1 | 0.03 |
| *Glaciecola* | 2 | 2 | 4 | 1 | 1 | 3 | 3 | 2 | **5** |  | - | - | - | - | - | - |
| NS3a marine group | **11** | **10** | **9** | **5** | **6** | **7** | **13** | **13** | **11** |  | **7** | **8** | 3 | 3 | **16** | **13** |
| *Polynucleobacter* | 3 | 2 | 2 | 4 | 4 | **5** | 0 | 0 | 0 |  | - | - | - | - | - | - |
| *Pseudomonas* | - | - | - | - | - | - | - | - | - |  | **6** | 3 | 1 | 0.4 | 0 | 0.03 |
| *Rubritalea* | 4 | **5** | 2 | **8** | **9** | 4 | **5** | **9** | 4 |  | 2 | 3 | 2 | 3 | 4 | **5** |
| *Vibrio* | - | - | - | - | - | - | - | - | - |  | **6** | **7** | 0.4 | 0.3 | 1 | 0.4 |
| *Burkholderiales Incertae Sedis* (u.g.) | 1 | 0.01 | 0.003 | **7** | 2 | 1 | 0 | 0 | 0 |  | 0.1 | 0.1 | **7** | **6** | 0.001 | 0 |
| *Cryomorphaceae* (u.g.) | 4 | 3 | 3 | 1 | 1 | 2 | **5** | 4 | **5** |  | 2 | 3 | 0.3 | 0.4 | **6** | **6** |
| *Flavobacteriaceae* (u.g.) | **10** | **13** | **9** | **6** | **8** | **5** | **13** | **14** | **12** |  | **6** | **9** | 3 | 4 | **12** | **11** |
| *Paracaedibacteraceae* (u.g.) | - | - | - | - | - | - | - | - | - |  | 0.3 | 0.3 | 3 | **6** | 0.003 | 0.01 |
| *Rhodobacteraceae* (u.g.) | 2 | 2 | 2 | **5** | **5** | 2 | 4 | 3 | 4 |  | 2 | 2 | 1 | 1 | **7** | **7** |
| *Burkholderiales* (u.g.) | 1 | 0.4 | 0.1 | **6** | 2 | 1 | 0 | 0 | 0 |  | 4 | 2 | **12** | 4 | 0.004 | 0 |
| *Bacteroidia* (u.g.) | 1 | 2 | 2 | 2 | **6** | **8** | 0.3 | 0.3 | 0.3 |  | - | - | - | - | - | - |

Additional file 4: Relative mean proportions (%) of the most abundant phyla and genera (≥5%) in the skin and gill microbiota of the different age groups of the seabass *Dicentrarchus labrax* and the seabream *Sparus aurata,* and in the water column (n=60 per species x age group for tissues; n=10 per species x age group for water). Taxa with a ≥5% relative mean proportion in a group are indicated in bold. Unknown genera are identified as u.g.
