# Supplementary material for: Effects of aging on the skin and gill microbiota of farmed seabass and seabream
Source: Anim Microbiome. 2021 Jan 12;3:10. doi: 10.1186/s42523-020-00072-2 (PMC7934244; doi:10.1186/s42523-020-00072-2)
Supplement: Supplementary file 5 — Additional file 5. [file 42523_2020_72_MOESM5_ESM.docx]

|  | Seabass | | | | | | | |  | Seabream | |
| --- | --- | --- | --- | --- | --- | --- | --- | --- | --- | --- | --- |
|  | Skin | | | | Gill | | | |  | Skin | Gill |
|  | Overall | EJ vs LJ | LJ vs MA | EJ vs MA | Overall | EJ vs LJ | LJ vs MA | EJ vs MA |  | J vs MA | J vs MA |
| Phyla |  |  |  |  |  |  |  |  |  |  |  |
| *Bacteroidota* | **5 (0.01)** | 2 (0.2) | **3 (0.01)** | 1 (0.4) | **25 (4^-10^)** | **5 (0.001)** | 2 (0.05) | **7 (0.001)** |  | **16 (9^-5^)** | **7 (0.01)** |
| *Cyanobacteria* | - | - | - | - | - | - | - | - |  | - | 4 (0.1) |
| *Proteobacteria* | **18 (2^-7^)** | **-6 (0.001)** | **2 (0.05)** | **-4 (0.001)** | **18 (9^-8^)** | **-5 (1^-5^)** | 1 (0.9) | **-5 (1^-5^)** |  | **8 (0.01)** | **19 (3^-5^)** |
| *Verrucomicrobiota* | **13 (7^-6^)** | **4 (0.0002)** | **5 (1^-4^)** | 1 (0.8) | **10 (0.0001)** | **3 (0.04)** | **-5 (0.001)** | -2 (0.1) |  | - | **20 (2^-5^)** |
| Total % of phyla variation | 100% | 67% | 100% | 33% | 100% | 100% | 33% | 67% |  | 100% | 75% |
| Genera |  |  |  |  |  |  |  |  |  |  |  |
| *Burkholderia-Caballeronia-Paraburkholderia* | - | - | - | - | - | - | - | - |  | 1 (0.3) | 0.04 (0.9) |
| *Glaciecola* | - | - | - | - | - | - | - | - |  | - | - |
| NS3a marine group | **11 (2^-5^)** | **4 (1^-4^)** | 0.2 (0.9) | **-4 (0.0001)** | 0.3 (0.7) | 0.1 (0.9) | 1 (0.8) | 1 (0.7) |  | 2 (0.1) | - |
| *Polynucleobacter* | - | - | - | - | 2 (0.1) | 0.003 (1) | 2 (0.2) | 2 (0.2) |  | - | - |
| *Pseudomonas* | - | - | - | - | - | - | - | - |  | **7 (0.01)** | - |
| *Rubritalea* | **4 (0.02)** | 1 (0.8) | -2 (0.1) | **-3 (0.02)** | **3 (0.05)** | -2 (0.2) | -1 (0.8) | **-2 (0.04)** |  | - | - |
| *Vibrio* | - | - | - | - | - | - | - | - |  | 0.3 (0.6) | - |
| *Burkholderiales Incertae Sedis* (u.g.) | - | - | - | - | **6 (0.01)** | **-3 (0.004)** | 0.9 (0.6) | -2 (0.1) |  | - | 1 (0.4) |
| *Cryomorphaceae* (u.g.) | - | - | - | - | - | - | - | - |  | - | - |
| *Flavobacteriaceae* (u.g.) | **13 (6^-6^)** | **4 (0.001)** | -1 (0.5) | **-5 (1^-4^)** | 0.7 (0.5) | 0.1 (0.9) | -1 (0.5) | -1 (0.6) |  | **7 (0.01)** | - |
| *Paracaedibacteraceae* (u.g.) | - | - | - | - | - | - | - | - |  | - | **17 (8^-5^)** |
| *Rhodobacteraceae* (u.g.) | - | - | - | - | **6 (0.003)** | 2 (0.3) | 2 (0.1) | **3 (0.002)** |  | - | - |
| *Burkholderiales* (u.g.) | - | - | - | - | **11 (5^-5^)** | -2 (0.3) | **5 (0.001)** | **3 (0.01)** |  | - | **15 (0.0002)** |
| *Bacteroidia* (u.g.) | - | - | - | - | **3 (0.05)** | 2 (0.2) | **-2 (0.04)** | -1 (0.8) |  | - | - |
| Total % of genera variation | 100% | 67% | 0% | 100% | 63% | 13% | 25% | 38% |  | 40% | 50% |

Additional file 5: Overall and pairwise comparisons of the relative proportions of the most abundant (≥5%) phyla and genera in the skin and gill microbiota of the seabass *Dicentrarchus labrax* and the seabream *Sparus aurata* across age groups (n=60 per species x age group x tissue). Variation in taxa proportion was assessed using Linear Mixed Effect models with age group as a fixed factor and sampling time as a random factor. For each linear model effect model test we report the F statistic and significance (P value). Significant differences are indicated in bold. EJ: early juveniles; LJ: late juveniles; MA: mature adults; J: juveniles.
