# Supplementary material for: Effects of aging on the skin and gill microbiota of farmed seabass and seabream
Source: Anim Microbiome. 2021 Jan 12;3:10. doi: 10.1186/s42523-020-00072-2 (PMC7934244; doi:10.1186/s42523-020-00072-2)
Supplement: Supplementary file 6 — Additional file 6. [file 42523_2020_72_MOESM6_ESM.docx]

|  | Seabass | | | | | | Seabream | | | |
| --- | --- | --- | --- | --- | --- | --- | --- | --- | --- | --- |
|  | Skin | | | Gill | | | Skin | | Gill | |
|  | EJ | LJ | MA | EJ | LJ | MA | J | MA | J | MA |
| **Biosynthesis** |  |  |  |  |  |  |  |  |  |  |
| Amine and Polyamine biosynthesis (L2) |  |  |  |  |  |  | 3 |  |  |  |
| Chlorophyll biosynthesis |  |  |  |  |  | 2 |  |  |  |  |
| Choline biosynthesis (L3) |  |  |  |  |  |  | 1 |  |  |  |
| Fatty acid biosynthesis (L3) |  |  |  |  |  |  |  | 1 |  |  |
| Folate biosynthesis |  |  |  |  |  | 1 |  |  |  |  |
| Hemiterpene biosynthesis |  |  |  |  |  | 2 |  |  |  |  |
| L-alanine biosynthesis |  |  |  |  |  | 1 |  |  |  |  |
| L-methionine biosynthesis |  |  |  |  |  |  |  | 1 |  |  |
| L-tyrosine biosynthesis |  |  |  |  |  | 1 |  |  |  |  |
| Metabolic regulator biosynthesis (L2) |  |  |  | 1 |  |  |  |  |  |  |
| NAD biosynthesis |  |  |  |  |  | 1 |  | 1 |  |  |
| Palmitate biosynthesis |  |  |  |  |  |  |  | 1 |  |  |
| Palmitoleate biosynthesis |  |  |  |  |  |  |  | 1 |  |  |
| Pyrimidine deoxyribonucleotide de novo biosynthesis |  |  | 1 |  |  | 1 |  |  | 1 | 1 |
| Purine nucleotide salvage |  |  |  |  |  |  |  | 1 |  |  |
| Secondary metabolite biosynthesis (L2) |  |  |  |  |  | 1 |  |  |  |  |
| Siderophore biosynthesis (L3) |  |  |  |  |  |  |  | 1 |  |  |
| Stearate biosynthesis (L3) |  |  |  |  |  |  |  | 1 |  |  |
| Thiamine biosynthesis |  | 2 |  |  |  |  |  |  |  |  |
| Ubiquinol biosynthesis |  |  |  |  |  | 4 |  |  |  |  |
| Unsaturated Fatty acid biosynthesis |  |  |  |  |  |  |  | 2 |  |  |
| **Degradation/Utilization/Assimilation** |  |  |  |  |  |  |  |  |  |  |
| Aldehyde degradation (L2) |  | 1 |  |  |  |  |  |  |  |  |
| Amine and Polyamine degradation (L2) |  |  |  |  |  |  | 1 |  |  |  |
| Autotrophic CO_2_ fixation |  |  |  |  |  |  |  |  |  | 1 |
| Chloroaromatic compound degradation |  |  |  |  |  | 1 |  |  |  |  |
| Denitrification |  |  |  |  | 1 |  |  |  |  |  |
| Galactose degradation |  |  |  |  | 1 |  |  |  |  |  |
| L-arabinose degradation |  | 1 |  |  |  |  |  |  |  |  |
| Nitrogen compound metabolism (L3) |  |  |  |  | 1 |  |  |  |  |  |
| Other |  |  |  |  |  |  |  | 1 |  |  |
| Purine nucleotide degradation (L3) |  |  |  | 1 |  |  |  |  |  |  |
| Sugar degradation (L3) |  |  |  | 1 |  |  |  |  |  |  |
| Sugar acid degradation (L3) |  |  |  |  |  |  | 1 |  |  |  |
| Toluene degradation (L3) |  |  |  |  |  |  | 2 |  |  |  |
| **Generation of Precursor Metabolites and Energy** |  |  |  |  |  |  |  |  |  |  |
| Aspartate superpathway (L2) |  |  |  |  |  |  |  | 1 |  |  |
| Fermentation of pyruvate (L3) |  |  |  | 1 |  |  |  |  |  | 1 |
| Fermentation to lactate |  |  |  |  |  | 1 |  |  |  |  |
| Glycolysis (L2) |  |  |  |  |  | 2 |  |  |  |  |
| Other |  |  |  |  |  | 1 |  |  |  | 1 |
| TCA cycle (L2) |  |  |  |  |  |  |  | 2 |  |  |
| **Macromolecule Modification** |  |  |  |  |  |  |  |  |  |  |
| Nucleic Acid Processing |  |  |  |  |  | 1 |  |  |  |  |
| Total | 0 | 4 | 1 | 4 | 3 | 20 | 8 | 14 | 1 | 4 |

Additional file 6: Significantly enriched pathways recovered from the skin and gill of the early juveniles (EJ), late juveniles (LJ) and mature adults (MA) of the seabass *Dicentrarchus labrax* and the juveniles (J) and mature adults (MA) of the seabream *Sparus aurata*. LEfSe tests were performed with a P value and LDA score cut-offs of 0.05 and of 2, respectively.
