# Supplementary material for: Effects of aging on the skin and gill microbiota of farmed seabass and seabream
Source: Anim Microbiome. 2021 Jan 12;3:10. doi: 10.1186/s42523-020-00072-2 (PMC7934244; doi:10.1186/s42523-020-00072-2)
Supplement: Supplementary file 7 — Additional file 7 PCoA plot computed using Bray-Curtis distances for water, skin and gills microbiota of the seabass Dicentrarchus labrax (A) and the seabream Sparus aurata (B) (n = 60 per species x age group for tissues; n = 10 per species x age group for water). Each dot represents a microbiome sample and is coloured by tissue/origin (skin, gill and water). [file 42523_2020_72_MOESM7_ESM.pdf]

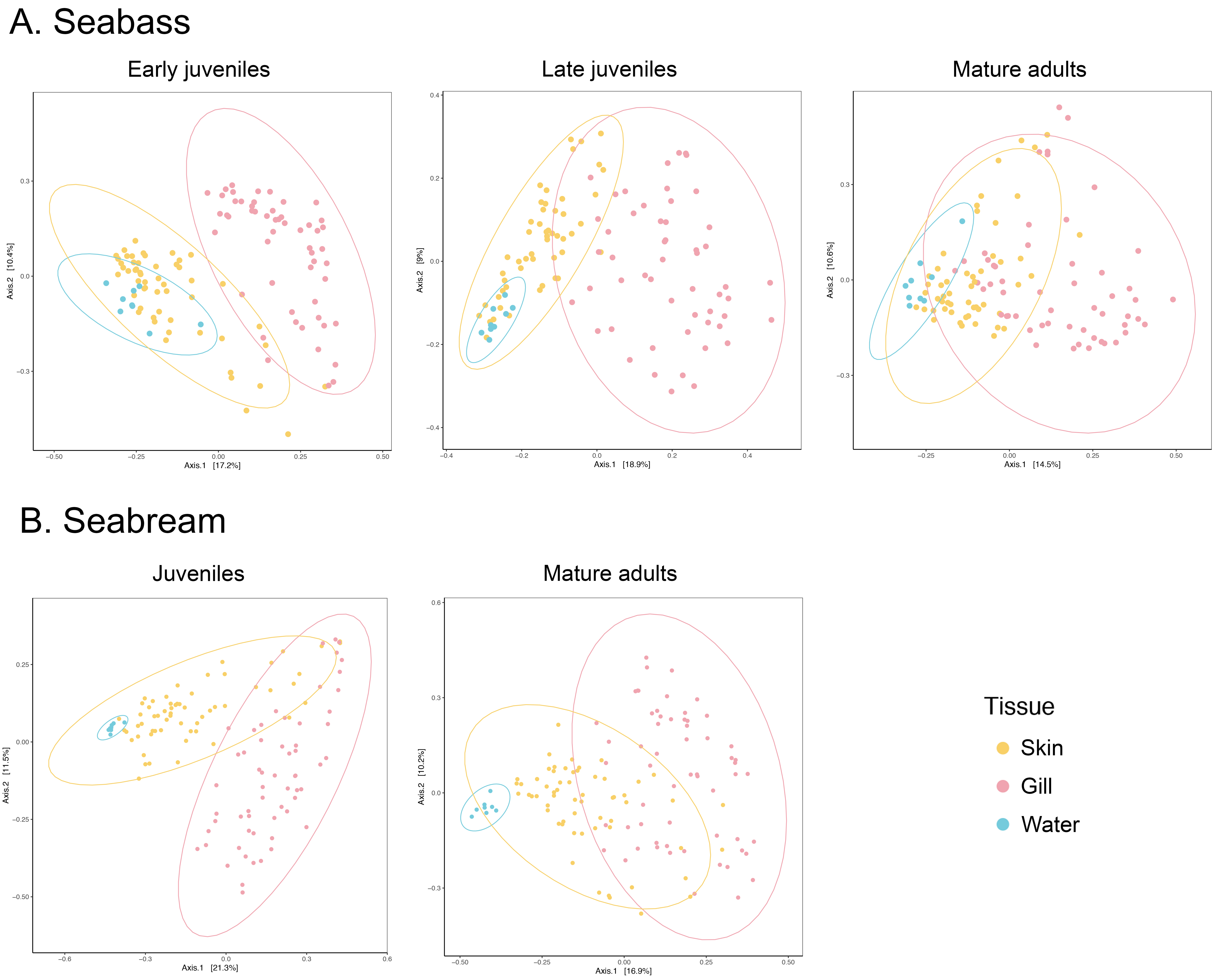

A. Seabass
Early juveniles
Late juveniles
Mature adults
Axis.1 [17.2%]
Axis.2 [10.4%]
Axis.1 [18.9%]
Axis.2 [9%]
Axis.1 [14.5%]
Axis.2 [10.6%]
B. Seabream
Juveniles
Mature adults
Axis.1 [21.3%]
Axis.2 [11.5%]
Axis.1 [16.9%]
Axis.2 [10.2%]
Tissue
Skin
Gill
Water
